# OmniNeo: a multi-omics pipeline incorporating proteomics and AI selection for neoantigen optimization in tumor immunotherapy

**DOI:** 10.3389/fimmu.2025.1727642

**Published:** 2025-12-17

**Authors:** Manman Lu, Yang Liu, Linfeng Xu, Yuan Gao, Peng Liu, Zhenhao Liu, Xiaoxiu Tan, Wenzhen Li, Yong Lin, Lanming Chen, Lunquan Sun, Lu Xie

**Affiliations:** 1 College of Food Science and Technology, Shanghai Ocean University, Shanghai, China; 2 Shanghai-Ministry of Science and Technology (MOST) Key Laboratory of Health and Disease Genomics, Shanghai Institute for Biomedical and Pharmaceutical Technologies, Shanghai, China; 3 Fudan Microbiome Center, State Key Laboratory of Genetic Engineering, Human Phenome Institute, and School of Life Sciences, Fudan University, Shanghai, China; 4 School of Health Science and Engineering, University of Shanghai for Science and Technology, Shanghai, China; 5 Xiangya Cancer Center, National Clinical Research Center for Geriatric Disorders, Xiangya Hospital, Central South University, Changsha, China; 6 Shanghai Institute for Biomedical and Pharmaceutical Technologies, School of Public Health, Fudan University, Shanghai, China

**Keywords:** neoantigens, multi-omics data, deep learning, tumor vaccine, immunotherapy

## Abstract

Neoantigen-based vaccines represent a promising approach in cancer immunotherapy, with the key to their effective clinical application lying in the precise identification of immunogenic neoantigens. Existing methods primarily focus on genomic variations, lacking integration of multi-omics data and essential filtering steps, which limits comprehensive assessment of immunogenicity and results in only a small subset of neoantigens capable of eliciting effective immune responses. Moreover, the complexity and poor portability further hinder the clinical applicability. To address these limitations, we developed OmniNeo, an automated multi-omics-based neoantigen discovery framework. 1) OmniNeo integrates whole-genome/exome sequencing (WGS/WES), transcriptomic, and proteomics data to simultaneously identify neoantigenic epitopes derived from SNVs/Indels, frameshift mutations, gene fusions, and non-coding region variations; 2) The pipeline incorporates a convolutional neural network-based model, OmniNeo-CNN along with multiple filtering mechanisms to quantify the immunogenicity and T-cell receptor (TCR) recognition potential of predicted neoantigen candidates through multiple features; 3) The workflow is built on nextflow, offering a one-stop, scalable, and portable solution for rapid and efficient neoantigen prediction. Finally, we demonstrated the practical application procedures of this workflow in potential tumor immunotherapy through case study analyses of liver cancer samples. The tool is freely accessible as an open-source resource via https://github.com/linfengxu/OmniNeo, https://zenodo.org/records/15340824.

## Introduction

Cancer is one of the leading causes of death globally, and tumor immunotherapy is emerging as a critical strategy to improve patient survival and potentially achieve a cure (1, 2). Neoantigens, as tumor-specific antigens expressed exclusively in cancer cells, are presented by major histocompatibility complex (MHC) class I and II molecules, referred to as human leukocyte antigen (HLA) in humans and specifically recognized by T-cell receptors (TCRs), may thereby trigger a *de novo* immune response (3, 4). Neoantigen-based immunotherapy can give rise to a range of effective treatment strategies, including tumor vaccines, immune checkpoint inhibitors (CPIs) combination therapies, and adoptive T-cell therapies. Such approaches have demonstrated promising efficacy in clinical trials for melanoma, hepatocellular carcinoma, colorectal cancer, pancreatic cancer, and lung cancer (5–7), and have even offered new hope for patients with “cold” tumors (8).

The precise identification and discovery of neoantigens is a core challenge in translating the related potential tumor immunotherapy strategy into clinical practice. Current mainstream prediction approaches primarily rely on genomic and transcriptomic data, with some incorporating deep learning and other computational tools to improve predictive accuracy. Standard workflows typically involve somatic mutation detection, MHC typing, MHC binding affinity prediction, and immunogenicity assessment. Various tools have been developed to address neoantigen discovery from different types of genomic alterations. For example, tools for detecting neoantigens derived from single nucleotide variants (SNVs), insertions and deletions (Indels) include TSNAD v2.0 (9), NeoPredPipe (10), pTuneos (11), and pVACview (12). Representative tools for identifying gene fusion derived neoantigens include NeoFuse and FusionNeoAntigen (13, 14), while PGNneo, IEAtlas were used to identify immune peptides from non-coding regions (15, 16). In addition, integrative pipelines such as nextNEOpi and ProGeo-neo2.0 are capable of handling multiple mutation types simultaneously (17–19). These tools have established a systematic framework from mutation annotation to candidate prioritization, facilitating the comprehensive characterization of the tumor immunogenic landscape.

However, due to the heterogeneity in omics data generation and resources, there still exist deficits in different pipelines for neoantigen discovery, and comprehensive one-stop station incorporating multiple omics data still in lack. Structural variations other than single mutations SNVs, and variation from non-coding regions can disrupt antigen processing and presentation mechanisms (APPM), thereby impairing the immune system’s ability to recognize and eliminate tumor cells (20). Inaccuracies in MHC typing and uncertainties surrounding peptide processing and presentation further undermine prediction reliability. Moreover, Current algorithms predominantly focus on MHC class I-restricted peptides, while overlooking the critical role of MHC class II-restricted peptides recognized by CD4+T cells mediated antitumor immunity (21), limiting comprehensive understanding of tumor-immune interactions. Therefore, there is an urgent need for more precise and systematic approaches to construct immunogenic epitope landscapes and overcome the challenge of limited neoantigen availability.

Emerging data-driven approaches, including systems vaccinology, and artificial intelligence, are continuously advancing the expansion of neoantigen maps and the development of personalized vaccine design (22). Among these, proteogenomic strategies that integrate next-generation sequencing (NGS) and mass spectrometry (MS) data provide an effective approach for the precise identification of immune peptides (3, 23). NeoDisc incorporated high-resolution immunopeptidomic data that directly detected MHC-bound peptides on the surface of tumor cells, revealing the repertoire of truly presented antigens (20). In addition, the introduction of nextflow workflow frameworks has greatly improved the efficiency of large-scale data processing while enabling visualization and reproducibility of complex computational steps. These advancements have paved the way for more sophisticated and efficient workflows in neoantigen identification.

In this study, we developed a multi-omics data-driven tumor neoantigen recognition pipeline, named OmniNeo. This pipeline integrates multiple key features that influence immunogenicity, including but not limited to transporter associated with antigen processing (TAP) transport efficiency, MHC binding affinity, gene expression levels, gene fusion and non-coding regions, presence of mutant peptides at the protein level and TCR recognition capability, enabling comprehensive identification of high-quality neoantigens. To demonstrate the case-study practices of OmniNeo, we applied it to mRNA vaccine and peptide vaccine designs from 4 cases of hepatocellular carcinoma, and verified through computational simulation that these vaccines can effectively induce anti-tumor immune responses. Finally, OmniNeo is implemented based on the nextflow framework, with all dependencies pre-installed in a containerized environment, effectively overcoming the deployment complexity and usability limitations of traditional tools. It supports rapid deployment across multiple platforms and enables efficient processing of high-throughput, large-scale datasets. In summary, this study provides a robust computational tool and practical framework that strongly supports the development of neoantigen-based mRNA and peptide vaccines.

## Materials and methods

### Data preparation

Whole-genome sequencing (WGS) data for four hepatocellular carcinoma patients, including both tumor and normal tissue samples were obtained from our previously collaborated and published work from the Chinese Human Proteome Project (CNHPP) (24). Transcriptome and mass spectrometry data were obtained from GEO (GSE124535) and iProX (IPX0000937000, http://www.iprox.org). We downloaded WES data and RNA-seq data from four other different solid tumor patients including three melanoma and one colon cancer from the NCBI SRA database (Bioproject IDs: PRJNA298310, PRJNA298330).

The human reference genome (hg38) and reference protein sequences were respectively downloaded from UCSC (https://hgdownload.soe.ucsc.edu/goldenPath/hg38/bigZips/latest/hg38.fa.gz) and the ensembl database (http://www.ensembl.org/). Subsequently, contaminated protein sequences were obtained from the common repository of adventitious proteins (cRAP) (https://www.thegpm.org/crap/) in FASTA format. The databases and URLs for downloading various data are listed in **Table 1**.

**Table 1:** Data sources.

Tumor type	Data type	Source	ID	Function
4 HCC patients	WGS data	CNHPP	GSE124535 IPX0000937000	mRNA vaccine and peptide vaccine design
	RNA-seq data	GEO		
	MS data	iProX		
3 melanoma patients 1gastrointestinal cancer patient	WES data/RNA-seq	NCBI SRA	PRJNA298310, PRJNA298330	Validation dataset
/	Human Reference Protein Database	Uniprot	/	Database Customized
	Contaminated laboratory protein sequences	cRAP		

**Figure 1:**
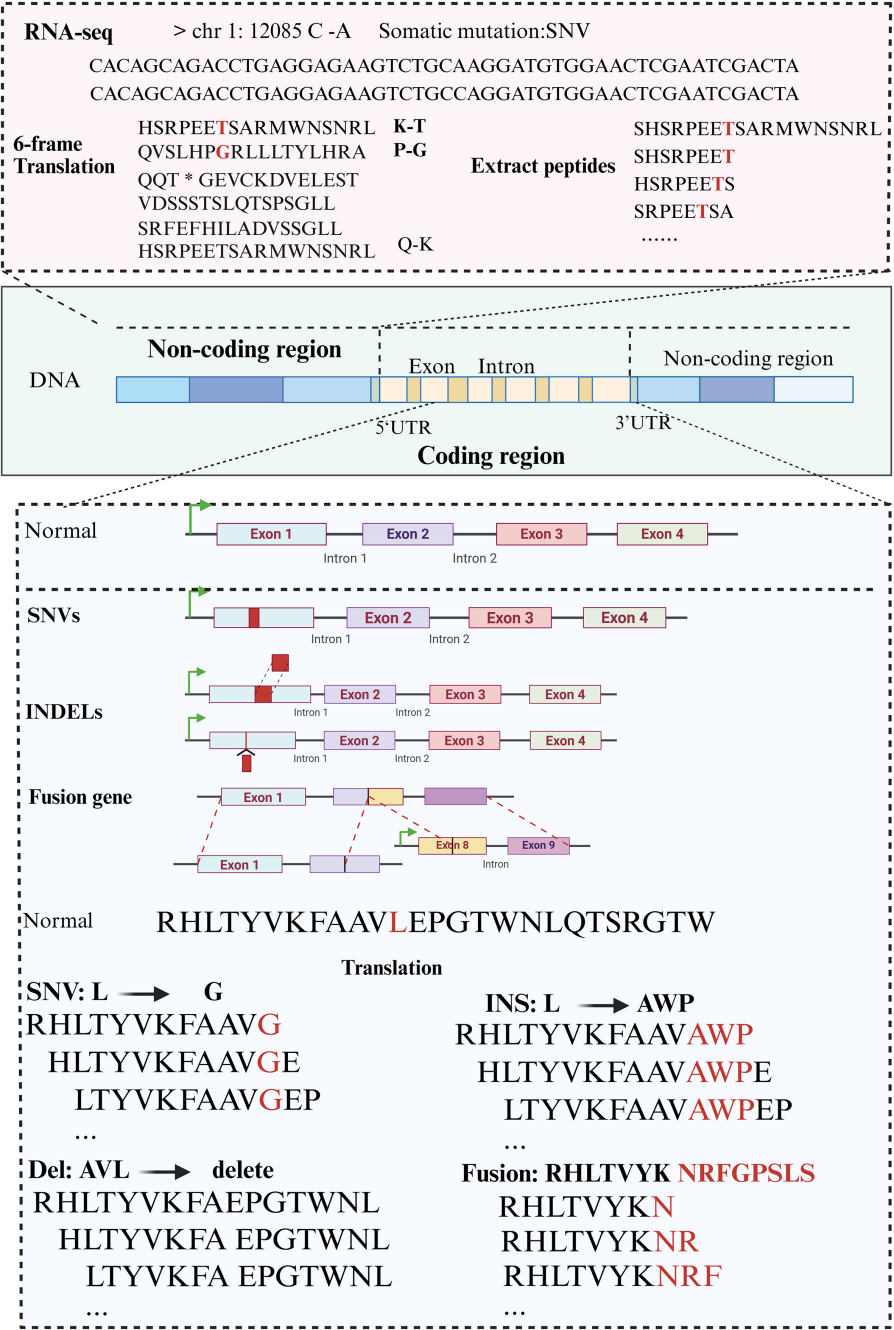
Identification and extraction of mutant peptides from diverse somatic mutation types. SNVs were directly mapped to protein references, while frameshift indels were translated from mutated cDNA. Peptides spanning the mutation site (m ± n) were defined as tumor-specific. For non-coding mutations, annotated by ANNOVAR, 100 bp flanking sequences were extracted and six-frame translated. Peptides without mutations were excluded, yielding mutant peptides from both coding and non-coding regions.

The raw WGS/WES data were cleaned using Trimmomatic (v0.39) and aligned to the human reference genome with BWA (25, 26). SAMtools (v1.10) was used to convert SAM to BAM format (27), and GATK was applied to remove duplicate reads and recalibrate quality scores (28). Somatic SNVs and InDels were detected using Mutect2 (29). RNA FASTQ files were processed with STAR-Fusion for fusion gene detection (30–32), and Kallisto (v0.46.2) was used to quantify transcripts per million (TPM) (33). HLA genotyping was inferred using OptiType and HLAminer (34, 35), among which OptiType has been demonstrated to achieve an accuracy of up to 97% (36). Mutations were annotated with ANNOVAR (37). For SNVs, genomic alterations can be directly applied to the proteomic reference, and for frameshift mutations within insertions or deletions, the mutated protein sequence is identified by translating the mutated cDNA sequence. For each mutation at position “m”, peptides from position m-n to m+n are defined as tumor-specific mutated peptides (**Figure 1**). For non-coding regions, somatic mutations based on RNA-seq data were annotated using ANNOVAR to identify mutations, such as intronic, splicing, and UTRs. Bedtools was employed to extract nucleotide sequences spanning 100 bases upstream and downstream of the mutation site (38), with reference bases replaced by mutant bases to construct mutation-containing sequences. The mutated nucleotide sequences were translated into novel protein sequences using the six-frame translation method, where stop codons were replaced with “*” and used as cleavage points to generate short peptides. Finally, peptides devoid of the mutation were removed, obtaining mutant peptide sequences from non-coding regions.

### Database customization and peptide identification

Identifying mutated peptides expressed at the protein level is a crucial step in neoantigen prediction. In this study, customized protein databases were constructed for each tumor sample, including human reference protein sequences, common laboratory contaminants, and cancer-specific protein sequences. MaxQuant software was used to search the MS/MS spectra (39), using a target-decoy search strategy, with decoy peptides generated using a reversed trypsin method. The MaxQuant parameters included N-terminal acetylation, methionine oxidation, and strict trypsin specificity, with a False Discovery Rate (FDR) set at 1%. Finally, cancer-specific mutated peptides were selected based on protein expression levels. Similarly, if matched immunopeptidomics data are available, a customized immunopeptide database also can be constructed through this module.

### Peptide binding affinity and transport efficiency calculation

NetMHCpan v4.1 and NetMHCIIpan v4.0 were used to calculate peptide binding affinity to human HLA-I and HLA-II molecules (40). NetMHCpan integrates 180,000 quantitative binding data and mass spectrometry-eluted ligand data for training. Its metric, “%Rank”, represents the binding strength between the peptide and MHC molecules. If %Rank ≤ 0.5, the peptide is considered a strong binder (SB) to MHC class I molecules; if 0.5 < %Rank ≤ 2, it is considered a weak binder (WB). For peptides binding to MHC class II molecules, a %Rank < 10 is used as the threshold (41). NetCTLpan was used to generate a comprehensive score by simulating C-terminal cleavage affinity and TAP transport efficiency (42). In addition, OmniNeo offers customizable filtering strategies, such as expression level > 0 nM, expression level > 33 nM as defined by TELSA, and similarity > 50% to the positive reference database. It also supports user-defined filtering parameters and peptide lengths.

### Immunogenicity calculation by deep learning and ranking of neoantigens

Another key factor in evaluating the immunogenicity of neoantigens is whether the peptide–major histocompatibility complex (pMHC) can be effectively recognized by TCRs, as this process determines its potential as a target for cytotoxic T cells (43). In this workflow, we developed a deep learning tool, OmniNeo-CNN, to evaluate the immunogenicity of candidate peptides (Only for class I peptides). The training dataset primarily consists of experimentally validated epitopes from the IEDB database, labeled as “positive” or “negative,” accounting for approximately 95% of the dataset. The remaining approximately 5% of negative samples were sourced from previously published non-immunogenic peptide datasets (44, 45). The collected data were further filtered, retaining only peptides consisting of 8–11 amino acids for model training. All HLA types were maintained at the 4-digit allele level, and neoantigen peptides with identical sequences but associated with different HLA alleles were treated as distinct neoantigens. Following data standardization, A total of 10,834 peptides were used for model training, with 4,106 positive samples and 6,728 negative samples. Given that the immunogenicity of neoantigens is influenced by multiple factors, we adopted one-hot encoding after comparing various encoding strategies. This approach incorporates key features, including TAP transport efficiency, MHC presentation, amino acid physicochemical properties, and MHC-antigen interactions, into a convolutional neural network module (**Supplementary Figure S1**). The physicochemical properties of amino acids were directly extracted from validated parameters in the AAindex database as input features. The database consists of three parts: AAindex1 (amino acid indices), AAindex2 (amino acid substitution matrices), and AAindex3 (statistical protein contact potentials). These indices cover six categories: α-helix and turn propensities, β-strand propensities, amino acid composition, hydrophobicity, physicochemical properties (e.g., polarity, charge, volume, accessibility), and other characteristics. In this study, we integrated multidimensional descriptors from AAindex to comprehensively characterize amino acids’ biophysical and biochemical properties, enhancing the model’s ability to learn protein sequence features.

The model was implemented in PyTorch and trained on a GPU when available. The network architecture comprises two parallel convolutional branches that process the MHC pseudosequence (34 amino acids) and the peptide sequence (11 amino acids), respectively. Each sequence is represented using both one-hot encoding (20 dimensions) and AAindex-based physicochemical features (22 dimensions), yielding a 42-dimensional feature vector at each amino acid position. The features extracted by the convolutional layers are concatenated with two global features (TAP binding score and NetMHCpan %Rank_EL) and subsequently passed to fully connected layers with dropout rates of 0.5 and 0.3 for binary classification. The model was trained using the Adam optimizer (learning rate = 0.0001, weight decay = 0.001) with a batch size of 256 for 50 epochs, with cross-entropy loss as the objective function. The dataset was split into training (80%) and validation (20%) sets, and model performance was evaluated using AUC, AUPR, accuracy, precision, and recall. All source code and datasets are available in a public GitHub repository (URL: *https://github.com/linfengxu/OmniNeo/blob/main/model/train_comparative.ipynb*). This tool is designed to reduce the false-positive rate in neoantigen prediction and to further narrow the set of immunogenic peptides requiring experimental validation.

### Automated implementation of the computational workflow

The OmniNeo fully automated neoantigen identification pipeline is built using nextflow (46), a workflow tool that enables tasks to be executed across multiple computing infrastructures in a portable manner, ensuring the pipeline’s portability, scalability, and reproducibility across different environments. To run the pipeline, users only need to provide a file containing the absolute paths to the WES/WGS and RNA-seq FASTQ data for the tumor and matched normal samples. When proteomic mass spectrometry data is available, its absolute path can also be included in the input file to enable the execution of the protein-level filtering and identification module based on the aforementioned customized database. For detailed execution commands, please refer to the GitHub repository. Then, output files include several analysis steps: variant annotation, neoantigen filtering and selection, and immunogenicity calculation. The final output is a high-quality list of neoantigen peptides. All necessary dependencies, including Python scripts required for downstream analysis, have been pre-installed through Conda environments and Singularity container functionality, simplifying the environment setup. Users can download the relevant reference files and example samples from the GitHub repository at https://github.com/linfengxu/OmniNeo. The pipeline is designed with modularity, making it user-friendly even for those with limited bioinformatics expertise, thus enhancing the accessibility and usability of neoantigen identification.

### Computer simulation of immune response

C-ImmSim (https://150.146.2.1/C-IMMSIM/index.php) was applied to integrate key principles of immunology, including neoantigen diversity (47), MHC restriction, thymic education of T cells, neoantigen processing, clonal selection, and immune memory. It models the immune response to various neoantigens, such as bacteria, viruses, allergens, or tumor cells, by simulating both cellular and humoral mechanisms. C-ImmSim is an agent-based model implemented on a three-dimensional periodic lattice that reconstructs both cellular and humoral immunity at the cellular scale. Using discrete time steps, the model updates cell proliferation, migration, apoptosis, and memory formation to capture the temporal dynamics of immune responses. Compared with conventional vaccine development, integrating immunoinformatics-driven multi-epitope design with C-ImmSim-based simulation and pre-evaluation can reduce cost and timelines while enabling high-throughput screening and optimization of candidate epitopes and multi-epitope formulations. Based on the algorithm of the computer simulation tool C-ImmSim for immune response, we calculated and evaluated the designed mRNA vaccines to predict the immune response efficacy against the tumors. We additionally obtained experimentally validated “negative” epitopes from NEPdb as the negative control (45). The negative epitope construct was: ASIRNANLY-AAY-VVNPIIYFY-AAY-VSDGFTAVM-AAY-VSDMSLSLS-AAY-CTDTYMLEL-AAY-YTSEHAASV-AAY-SLAPLSPRV-AAY-MLFLRFCYI-AAY-SLSTSLSSVL-AAY-SLSSVTLLL.

### Design and optimization of secondary and tertiary structures of vaccine sequences

We prioritize the selecting of candidate peptides with high immunogenicity for the design of mRNA vaccines, peptide vaccines, and T-cell immunotherapies targeting neoantigen reactivity. Similar to natural mRNA, the design of mRNA vaccines requires the inclusion of five components from the 5’ to 3’ end: the 5’ cap, 5’ untranslated region (UTR), coding sequence (CDS), 3’ UTR, and poly (A) tail. Considering the susceptibility of mRNA to non-enzymatic degradation and the host cell’s mRNA degradation system, we optimized the vaccine sequence to enhance its utilization of the eukaryotic protein translation machinery and ensure sufficient half-life to achieve the desired expression levels and duration. For peptide vaccines, we utilized PSIPRED 4.0 to predict the secondary structure and 3DPro to model the tertiary structure (48, 49). The initial models were optimized using the GalaxyRefine web server to improve structural rationality and stability (50). The refined structures were validated using the Ramachandran plot to assess their structural quality. For TCR-pMHC docking simulations, we employed in-house iTCep and GRATCR to predict potential TCR sequences that may react with the neoantigen (51, 52). Subsequently, TCRmodel2 was used to model and optimize the TCR-pMHC complex (53), yielding high-resolution structural models. Finally, PyMOL was utilized to visualize the 3D structures and docking patterns for further analysis and validation (https://www.pymol.org/).​​

## Result

### OmniNeo-CNN model for immunogenicity prediction

To identify key features influencing the immunogenicity of neoantigens, we compared immunogenic and non-immunogenic peptides in terms of TAP transport efficiency and proteasomal C-terminal cleavage. The results showed a significant difference in TAP transport efficiency between the two groups, whereas no significant difference was observed in proteasomal cleavage efficiency (**Supplementary Figures S2A, B**), suggesting that TAP transport efficiency may play a crucial role in the development of immunogenicity. In addition, previous studies have shown that aromatic and hydrophobic amino acid residues within peptides can enhance their binding affinity to TCRs (54). Based on the above findings, we incorporated TAP transport efficiency, amino acid physicochemical properties, and HLA binding affinity into the construction of the OmniNeo-CNN model for immunogenicity prediction. The dataset was randomly divided into training (60%), validation (20%), and independent test (20%) sets. Model performance was evaluated using receiver operating characteristic (ROC) curves and the area under the precision-recall curve (AUPR). On the independent test set, OmniNeo-CNN achieved an AUC of 0.88 and an AUPR of 0.81 (**Supplementary Figures S2C, D**), demonstrating strong robustness and the ability to accurately distinguish truly immunogenic neoantigens.

### Overview of the OmniNeo pipeline for neoantigen identification

**Figure 2:**
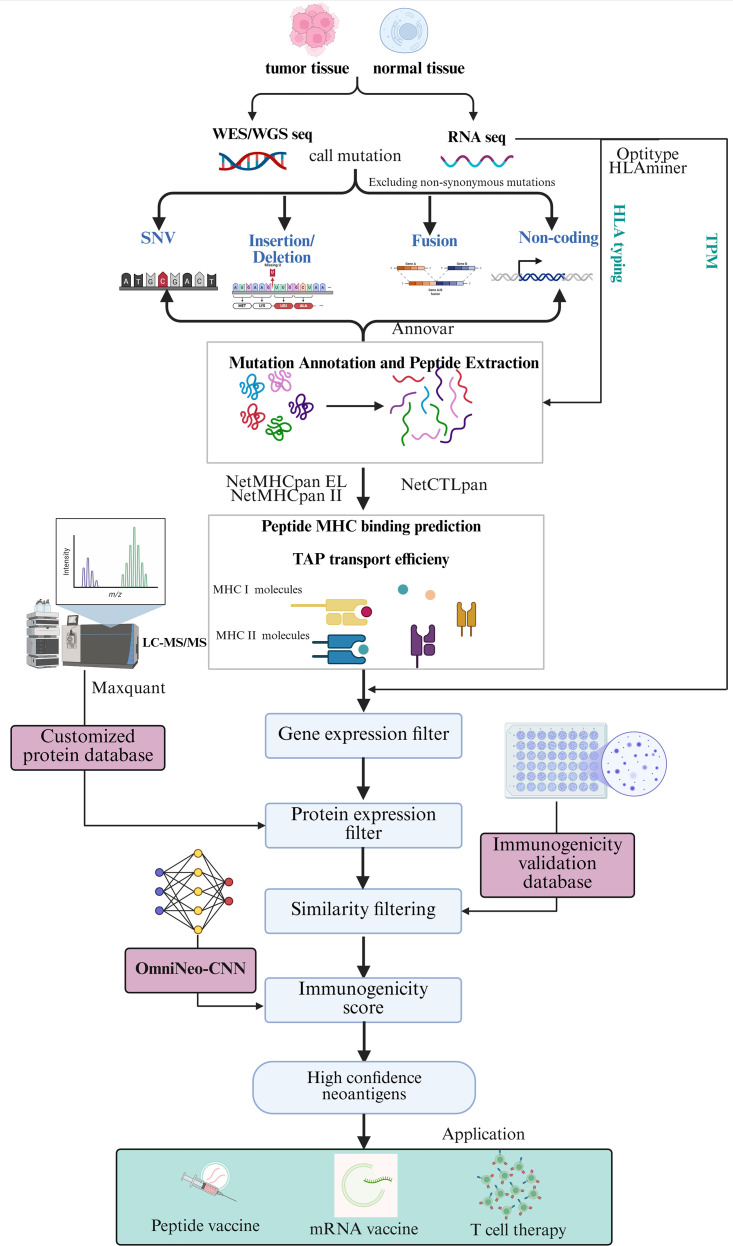
Overview of OmniNeo workflow. Starting from paired tumor/normal WES/WGS and tumor RNA-seq data, OmniNeo performs variant annotation, HLA typing, and peptide generation. Predicted peptides are filtered using multi-omics evidence, including RNA expression, mass spectrometry validation, and curated databases. Final candidate peptides are scored for immunogenicity using OmniNeo-CNN. The workflow integrates diverse neoantigen features to enable accurate and comprehensive neoantigen prioritization.

OmniNeo is a multi-omics-based neoantigen identification tool that provides an easy-to-use and reproducible workflow. The steps involved in the OmniNeo workflow are detailed below (**Figure 2**). Firstly, the pipeline begins with FASTQ data from paired tumor and normal WES/WGS, along with tumor RNA-seq data. Annotated mutations in both coding and non-coding regions, HLA allele information, and RNA quantitative expression are obtained. The mutation types primarily focus on variants that “directly alter the amino acid sequence and can immediately enter the presentation pathway,” such as missense mutations, frameshift mutations, gene fusions, and non-canonical translation-derived mutations, with the aim of ensuring the rationality of the candidate space and the controllability of computational costs. The second step involves HLA typing and TAP transfer efficiency calculation. HLA typing and TAP transfer efficiency are calculated using NetCTL, NetMHCpan (v4.1) and NetMHCIIpan (v4.0) to predict all possible 8~11-mer peptides presented by HLA-I molecules and 15~18-mer peptides presented by HLA-II molecules. The third step is to integrate multi-omics evidence to filter neoantigens, including transcriptional-level filtering, proteomics-level filtering, and positive standard database filtering. The fourth step was immunogenicity recognition (OmniNeo-CNN), where the validated peptides were subjected to immunogenicity calculation and top-ranked epitopes were considered the most immunogenic neoantigens. Compared with other published workflows (**Table 2**), the OmniNeo workflow incorporates a wider range of neoantigen characteristics into its computational prediction process, providing a more advanced and comprehensive set of functionalities.

**Table 2:** A comparative analysis of the functionalities between the OmniNeo pipeline and other established pipelines.

Pipelines	Input data	Neoantigen types	MHC restriction	MHC typing	MHC binding	Proteomic validation	Prioritization	Whether to support one-stop	Reference (Pubmed ID)
pTuneos (2019)	WES-seq, RNA-seq	SNV/Indel	I	OptiType	NetMHCpan 4.0	No	machine-learning model-RefinedNeo score	No	(11)
neoFusion (2019)	RNA-seq	Fusion	I	OptiType/Netchop	NetMHCpan 4.0	No	Immunogenicity score	No	(13)
MuPeXI (2017)	WES-seq/WGS-seq, RNA-seq	SNV/Indel	I	OptiType	NetMHCpan 3.0	No	HLA presentation abundance, Similarity	No	(68)
NeoPrePipe (2019)	Single and multi-region tumor VCF	SNV, Indel and stop-losses	I	POLYSOLVER	NetMHCpan	No	/	No	(10)
TruNeo (2020)	WES-seq/WGS-seq, RNA-seq	SNV, Indel, Fusion	I	Polysover, BWA-HLA	NetMHCPan 3.0	No	Deep learning-based model	No	(69)
neoANT-HILL (2020)	WES-seq/WGS-seq, RNA-seq	SNV, Indel, DEL	I	OptiType	MHCflurry+IEDB	No	/	No, Docker	(70)
TSNAD2.0 (2021)	WES-seq/WGS-seq, RNA-seq	SNV, Indel, Fusion	I	OptiType	/	No	DeepHLApan (immunogenic score >0.5)	Docker, Web	(9)
nextNEOpi (2021)	WES-seq/WGS-seq, RNA-seq	SNV, Indel, Fusion	I	/	NetMHCpan,MHCflurry	No	/	Yes, Nextflow	(19)
ProGeo-neo2.0 (2022)	WES-seq/WGS-seq, RNA-seq	SNV, Indel, DEL, Fusion	I, II	OptiType, HLAminer	NetMHCpan	Yes	/	No	(17)
PGNneo (2023)	RNA-seq	Non-coding region	I	OptiType	NetMHCpan 4.1	Yes	/	No, Docker	(15)
Neo-intline (2023)	WES-seq/WGS-seq, RNA-seq	SNV, Indel	I, II	/	NetMHCpan 4.0, NetMHCIIpan	No	Neoantigen probability score	No	(71)
OmniNeo(2025)	WGS-seq/WES-seq RNA-seq LC-MS/MS	SNV, Indel, Fusion, Noncoding region	I, II		NetMHCpan 4.1, NetMHCpan II 4.0	Yes	Neoantigen probability score	Yes, Nextflow	/

### Analysis of clinical HCC samples with OmniNeo workflow

**Figure 3:**
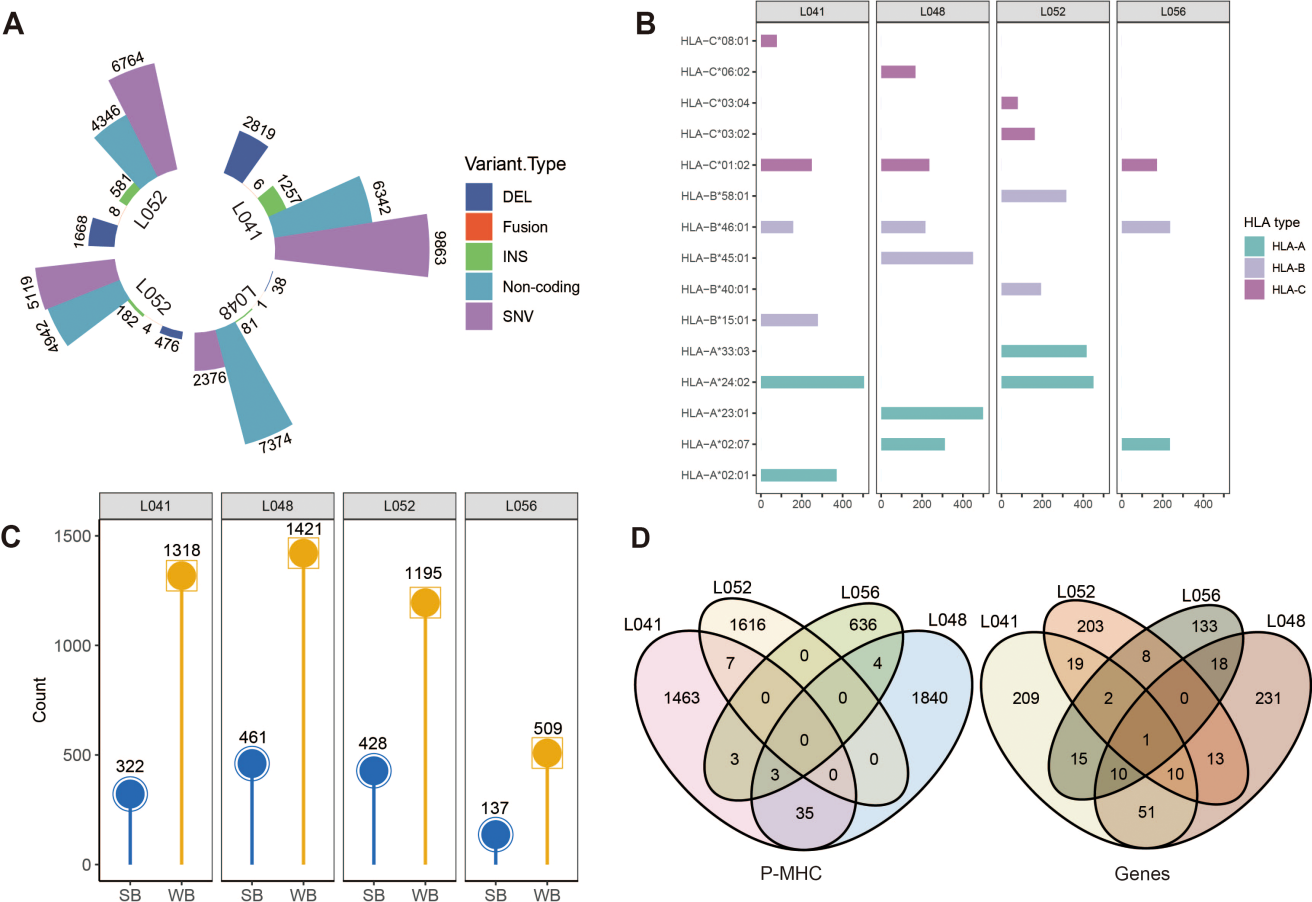
Statistical analysis of neoantigen prediction results for four HCC patients. **(A)** Statistics of mutation sites in the four HCC patients. **(B)** Calculated HLA genotypes and the number of peptides bound to HLA alleles. **(C)** Binding status of neoantigens, SB for strong binding, WB for weak binding. **(D)** p-HLA peptides burden and related genes overlap among the four patients.

We applied the OmniNeo pipeline to paired tumor/normal whole-genome sequencing, RNA-Seq, and LC-MS/MS datasets from four hepatocellular carcinoma (HCC) patients and performed statistical analysis of the prediction results, as shown in **Figure 3**. For each patient, we identified 2,376 to 9,863 SNVs, 81 to 1,257 insertion variants, 38 to 2,819 deletion variants, and 1 to 8 fusion variants. A total of 31,243 mutations from coding regions were identified across the four patients (**Figure 3A**). Based on RNA-seq data, each patient had 4,346 to 7,342 mutations, with a total of 23,004 mutations identified in non-coding regions (**Figure 3A**). We further used Optitype and HLAminer to predict HLA genotyping from RNA-seq FASTQ files, identifying 15 unique HLA-I alleles, 3 unique HLA-II alleles. Subsequently, NetMHCpan 4.1 and NetMHCIIpan were used to calculate peptide-HLA binding affinity, identifying 646 to 1,882 candidate neoantigens presented by HLA-I molecules and 40 to 737 candidate neoantigens presented by HLA-II molecules. Different patients exhibited preferences for binding to different HLA alleles (detailed filtering data of class I peptides are presented as an example). Patient L041 showed a strong preference for HLA-A0201 (n=371) and HLA-A2402 (n=505), while L048 showed a preference for HLA-A2301 (n=499) and HLA-B4501 (n=450). Patient L052 preferred HLA-A2402 (n _=_ 451) and HLA-A3303 (n=417), and L056 showed strong preference for HLA-A0207 (n=236) and HLA-B4601 (n=237) (**Figures 3B, C**). The HLA typing of each patient determines the tumor-specific neoantigen repertoire and T-cell specificity. Each patient has a unique HLA genotype (**Figure 3B**). Additionally, we observed that the same neoantigens generated by the same genes could be presented by different HLA alleles and exist in different patients (**Figure 3D**), suggesting the potential for shared neoantigen targets, which may have broader applicability across different patients.

### Computation and screening for shortlisting T cell epitopes

The functional importance of tumor neoantigens can be attributed to multiple factors (55). Merely considering tumor variations, HLA typing, and HLA binding affinity may lead to a large number of candidate peptides, many of which are false positives, hindering clinical validation. In the OmniNeo workflow, we have integrated four filtering methods that meticulously account for a multitude of biological processes, thereby enhancing the identification and selection of neoantigens with potentially high immunogenicity. 1) At the gene expression level, OmniNeo analyzed RNA-seq data and calculated expression levels, retaining 4,505 HLA class I gene-expressed candidate neoantigens and 3,090 HLA class II candidate neoantigens. 2) At the protein level, a custom database was constructed for each tumor sample, including human reference protein sequences, common laboratory contaminant protein sequences, and cancer-specific protein sequences. OmniNeo identified 2,339 mutant peptides expressed at the protein level through mass spectrometry. 3) TESLA investigated key features influencing peptide immunogenicity, including high binding affinity, high tumor abundance, high binding stability, and peptide recognition. These features were shown to effectively enrich for immunogenic peptides. In this study, we focused on two readily quantifiable features, binding affinity and tumor expression, and empirically selected the thresholds (binding affinity < 34 nM and tumor expression > 33 TPM) based on TESLA’s recommendations, aiming to narrow the candidate space and improve computational efficiency (56). The thresholds were applied to the OmniNeo workflow, ultimately identifying 127 candidate neoantigens. 4) OmniNeo established a target sequence repository comprising 2,357 experimentally validated peptides (The data were obtained from a positive neoantigen database established in house by our research group previously, as well as the Immune Epitope Database) (57). 5) From a pool of 127 candidate sequences, 47 highly credible peptides were identified through BLASTdb filtering, exhibiting substantial homology to immunogenic peptides. 6)The immunogenicity of potential neoantigens was calculated using OmniNeo-CNN, and 31 high-quality neoantigens were retained as potential target candidates for vaccine development (**Supplementary Figure S3**, **Supplementary Table S1**).

### Validation cohort from published studies

To evaluate the performance of the OmniNeo workflow, we applied it to data from three melanoma patients and one rectal cancer patient, whose neoantigen profiles were confirmed to include 14 experimentally validated neoantigen sites (58, 59). Using WES-seq and RNA-seq data from these four patients, we identified 9 of the 14 neoantigen sites, as shown in supplementary **Supplementary Table S2**. The prediction results of OmniNeo were compared with those of three other pipelines [pVACseq (60), TSNAD2.0 (61), and NeoPredPipe (10)]. The results showed that the experimentally validated neoantigen sites predicted by OmniNeo ranked higher than those identified by the other three pipelines. These results demonstrate that the OmniNeo workflow exhibits superior sensitivity in identifying truly immunogenic neoantigens and can effectively enhance screening efficiency.

### Further application scenarios

#### Case 1: design and immune validation of HCC mRNA vaccines

Once the prioritization and selection range of candidate neoantigens are determined (**Supplementary Table S2**), they can be utilized for tumor vaccine development and subsequent clinical cancer therapy. Typically, after intramuscular injection, mRNA vaccines are taken up by muscle cells, where they produce corresponding peptides. The synthesized peptides need to be secreted and then taken up by antigen-presenting cells (APCs) to further elicit an immune response. Therefore, we have incorporated three additional immune factors in vaccine design. One is a signal peptide, which guides the secretion of the synthesized peptides into the extracellular space. The second is the ligand of CD40, which acts as an adjuvant to enhance the uptake of synthesized peptides through the binding of CD40L on APCs to CD40 receptors. The third is the MHC-I-targeting domain (MITD), which directs the transport of synthesized CTL epitopes to the MHC-I region of the endoplasmic reticulum (62). Additionally, to save costs and enhance vaccine efficacy, we designed mRNA vaccine sequence capable of encoding multiple candidate neoantigens and added neoantigen linkers to link multiple neoantigen sequences. Furthermore, at the 5′ end, we added 5′m7G cap and β-globin 5′UTR, as well as α-globin 3′UTR and 120-150bp poly(A) tail to improve mRNA vaccine stability. Finally, the complete theoretical construct of the neoantigen mRNA vaccine for one personalized hepatocellular carcinoma patient (the top 10 prioritized neoantigens from patient L052) is assembled in the following order from the N-terminus to the C-terminus, as an example:

*5′m7G Cap – 5′-UTR of β-globin – GCCACCAUG (Kozak Sequence) – MDAMKRGLCCVLLLCGAVFVSPS (t-PA secretary signal peptide) – HIS tag (GTGGGGSHHHHHHGGMASMTGGQQQQMGG GGGSSR) – CD40L sequence (aa 116–261 extracellular domain) (as adjuvant) – GPGPG – neoantigen (IFSPGFFVGFL) – AAY (linker) – IFSPGFFVGF – AAY – FSPGFFVGF – AAY – DIFSPGFFVGF – AAY –QTAGPGGSR -AAY – VPGACNPSHLR – AAY – FFPSSPPNK – AAY – AEPPPFSGP– AAY – EAWASSLLIPW– AAY – TAGPGGSRL– AAY – MITD sequence – Stop codon – a-globin 3′-UTR – poly(A) tail*.

The C-ImmSim server was utilized to computationally evaluate the potential immune response triggered by this mRNA vaccine, while also performing negative controls based on experimental data from immunogenicity validation. The simulation evaluates the potential of the designed vaccine to elicit an immune response based on key indicators: the peak expansion of CD8+ CTLs reflects the effectiveness of the cytotoxic response; sustained activation of B cells, DCs, and NK cells indicates coordination between innate and adaptive immunity; activation and memory formation of CD4+ Th cells suggest strong immune memory; and significant increases in immunoglobulins and cytokines further support robust humoral and cellular immune responses.

**Figure 4:**
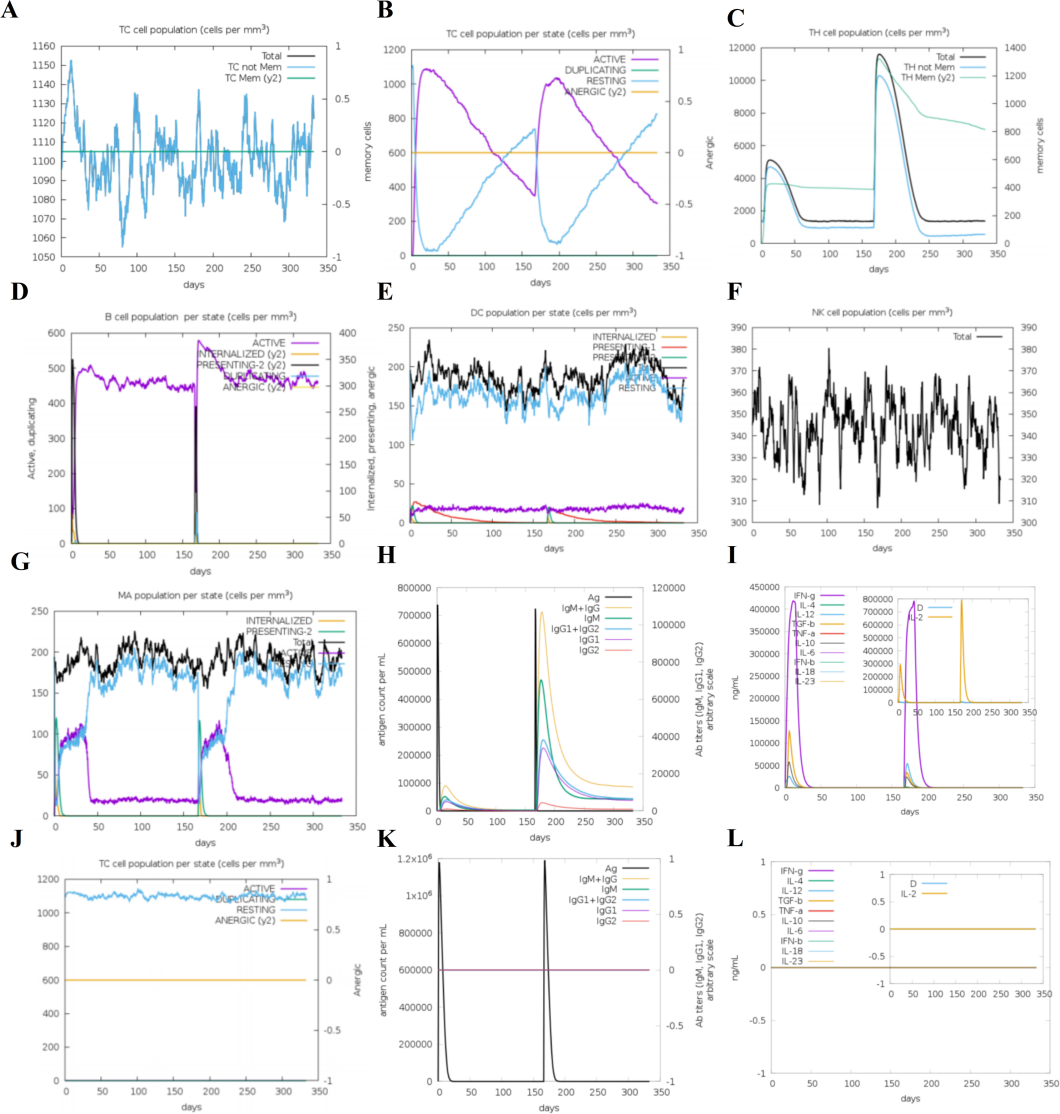
In silico immune simulation studies in response to mRNA vaccine using C-ImmSim. **(A)** Antigen and immunoglobulins. **(B)** Activated/effector CD8 CTLs exhibit the characteristic expansion–contraction–steady-state kinetics, with higher peaks and faster responses following booster immunization. **(C)** CD4 Th cell activation and memory: the sustained plateau above baseline indicates the establishment of durable helper function and immunological memory. **(D–G)** (B cells/dendritic cells DC/natural killer cells NK): The clonal expansion and maintenance of B cells correlate with the subsequent antibody curve; sustained activation of DCs indicates effective antigen presentation and co-stimulation; the increase in NK cells suggests the involvement of the innate immune arm in coordination. **(H)** A class switch from IgM to IgG occurs, with higher peaks and a larger AUC following booster immunization, consistent with a high-quality humoral response and affinity maturation. **(I)** Cytokines show a pulsatile increase after each immunization, with earlier and higher peaks following the second/third immunizations, reflecting an effective amplification of the Th1/CTL axis. **(J–L)** The negative control results, no significant induction of immunoglobulins was observed; IFN-γ and IL-2 levels remained at baseline levels; T cells did not undergo large-scale expansion, nor did they form or maintain an immune memory pool. These results indicate that the construct lacks immunogenicity.

The results of the immune response simulation indicate that, following vaccination, the population of cytotoxic T cells (CTL) is expected to increase, with activated CD8+ cytotoxic T lymphocytes peaking on day 25 post-immunization (**Figures 4A, B**). Additionally, B cells, dendritic cells (DCs), and natural killer (NK) cells are predicted to remain in an activated state throughout the immune response (**Figures 4D–G**). Notably, the CD4+ helper T cell (Th) population is expected to exhibit strong activation and establish immune memory, with memory cells anticipated to persist for several months (**Figure 4C**). During the induction of the immune response, high levels of immunoglobulins, including IgM+IgG, IgM, IgG, and IgG1+IgG2, are expected to be induced (**Figure 4H**). Furthermore, the vaccine is predicted to promote cytokine secretion, with significant increases in IFN-γ and IL-2 levels after each dose (**Figure 4I**). In contrast, the control vaccine, constructed with non-immunogenic peptides (negative data obtained from the NEPdb database), is expected to fail to induce immunoglobulins or cytokines, thereby not effectively triggering a T cell response (**Figures 4J–L**). Overall, the C-ImmSim immune simulation demonstrated that the multi-epitope mRNA vaccine we designed may have a strong potential to elicit robust immune responses against tumors.

#### Case 2: design and 3D simulation of multi-target HCC vaccines for cancer therapy

In this case, based on the results from patient L041 as an example, we constructed and evaluated a multi-epitope peptide vaccine (PepVaccine), demonstrating a systematic vaccine design strategy encompassing neoantigen selection, structural modeling, and computational immune simulation validation.

**Figure 5:**
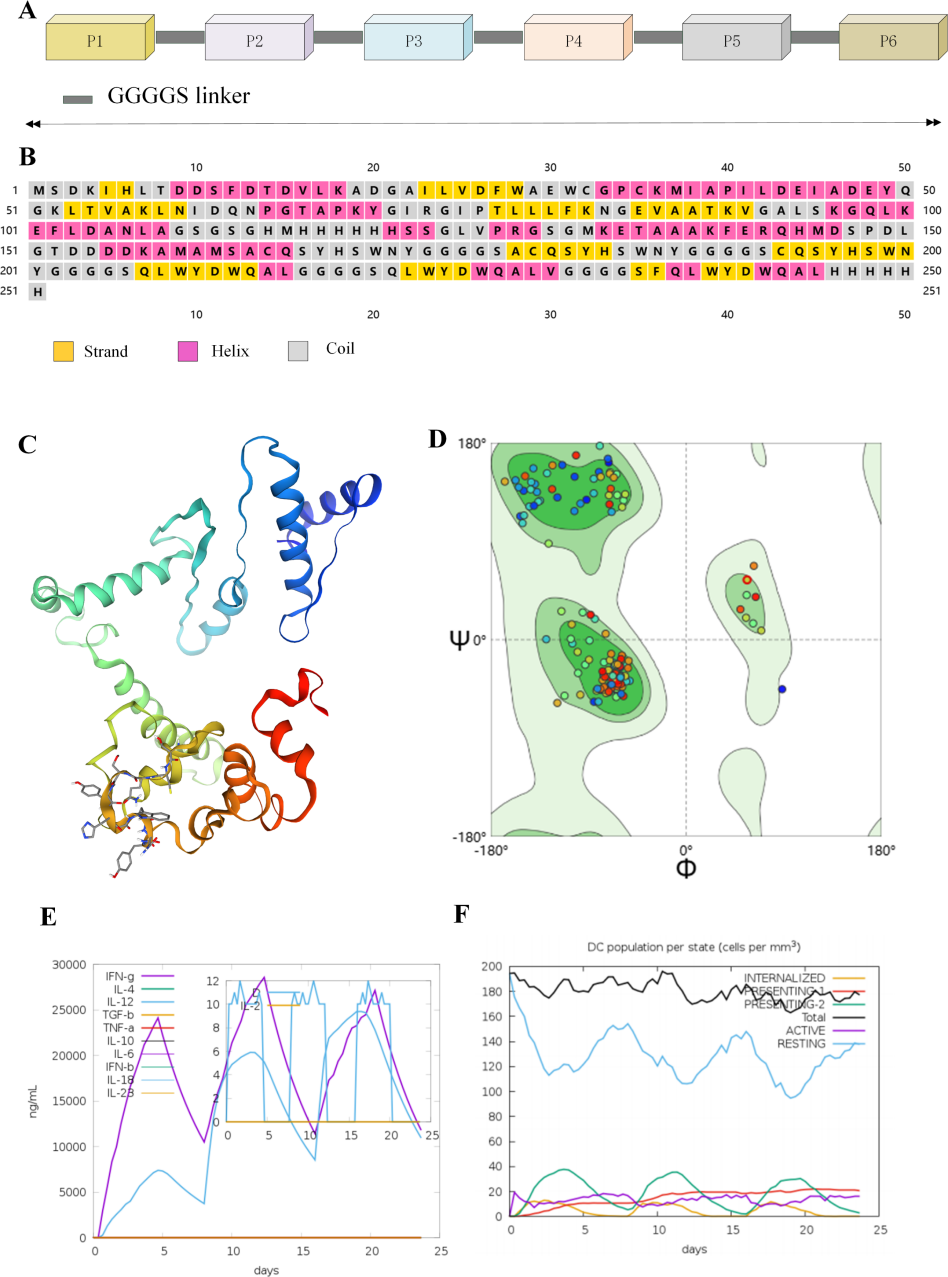
The secondary structure, optimized tertiary structure, and immune simulation experiments of the PepVaccine. **(A, B)** Secondary structure of the vaccine was predicted by the PSIPRED server. **(C)** The optimized tertiary structure of the PepVaccine was predicted by the Galaxy WEB server. **(D)** Ramachandran diagrams for the PepVaccine. **(E, F)** In silico immune simulation studies in response to PepVaccine using C-ImmSim.

The PepVaccine consists of six epitope peptides, which were linked using GGGGS flexible linkers to preserve the independent antigenicity of each epitope and enhance the structural stability of the overall conformation. First, based on the PSIPRED server, we predicted the secondary structure of the PepVaccine (**Figure 5A**), which is composed of 36.3% α-helix, 23.1% β-strand, and 40.6% random coil, indicating good folding potential and structural stability (**Figure 5B**). The 3D structure model of the PepVaccine was initially designed using the 3Dpro server and optimized using the Galaxy WEB server to refine the MP3RT vaccine model (**Figure 5C**). The higher GDT-HA value and lower MolProbity score indicate better model quality (**Table 3**). The optimized vaccine model, model 5, was used for further study. Further analysis using the Ramachandran plot also demonstrated a significant improvement in the percentage of residues in the core region of the optimized model, with the rama favored score increasing from 90.4 to 97.2 (**Table 3**), indicating a substantial improvement in structural stability and rationality, providing a reliable foundation for subsequent immunological studies. (**Figure 5D**). Finally, human computational immune simulations confirmed that the peptide vaccine effectively activated T lymphocytes and B lymphocytes, generating high levels of cytokines such as IFN-γ and IL-2, as well as antibodies (**Figures 5E, F**). This case validates the feasibility of neoantigen-driven multi-epitope peptide vaccines in personalized cancer immunotherapy and provides a standardized, reproducible framework for precision vaccine design.

**Table 3:** The peptide vaccine model parameters refined. .

Model	GDT-HA	RMSD	MolProbity	Clash score	Poor rotamers	Rama favored
Initial	1	0	3.563	113.2	4.7	90.4
MODEL 1	0.9143	0.504	1.935	15.6	0.5	96.4
MODEL 2	0.8994	0.538	1.971	15.6	0.5	96
MODEL 3	0.9213	0.492	1.962	16.6	0.5	96.4
MODEL 4	0.9094	0.505	1.943	13.4	0.5	95.6
MODEL 5	0.9143	0.513	1.843	15.6	0	97.2

Comparison of the initial and refined models (MODEL 1–5) based on GDT-HA, RMSD, MolProbity score, clash score, poor rotamers, and Ramachandran favored regions. Our refined MODEL 5 demonstrates the highest overall structural quality with the lowest MolProbity score, no poor rotamers, and the highest Ramachandran favored percentage.

#### Case 3: structure-guided identification of neoantigen specific TCRs for therapeutic engineering

**Figure 6:**
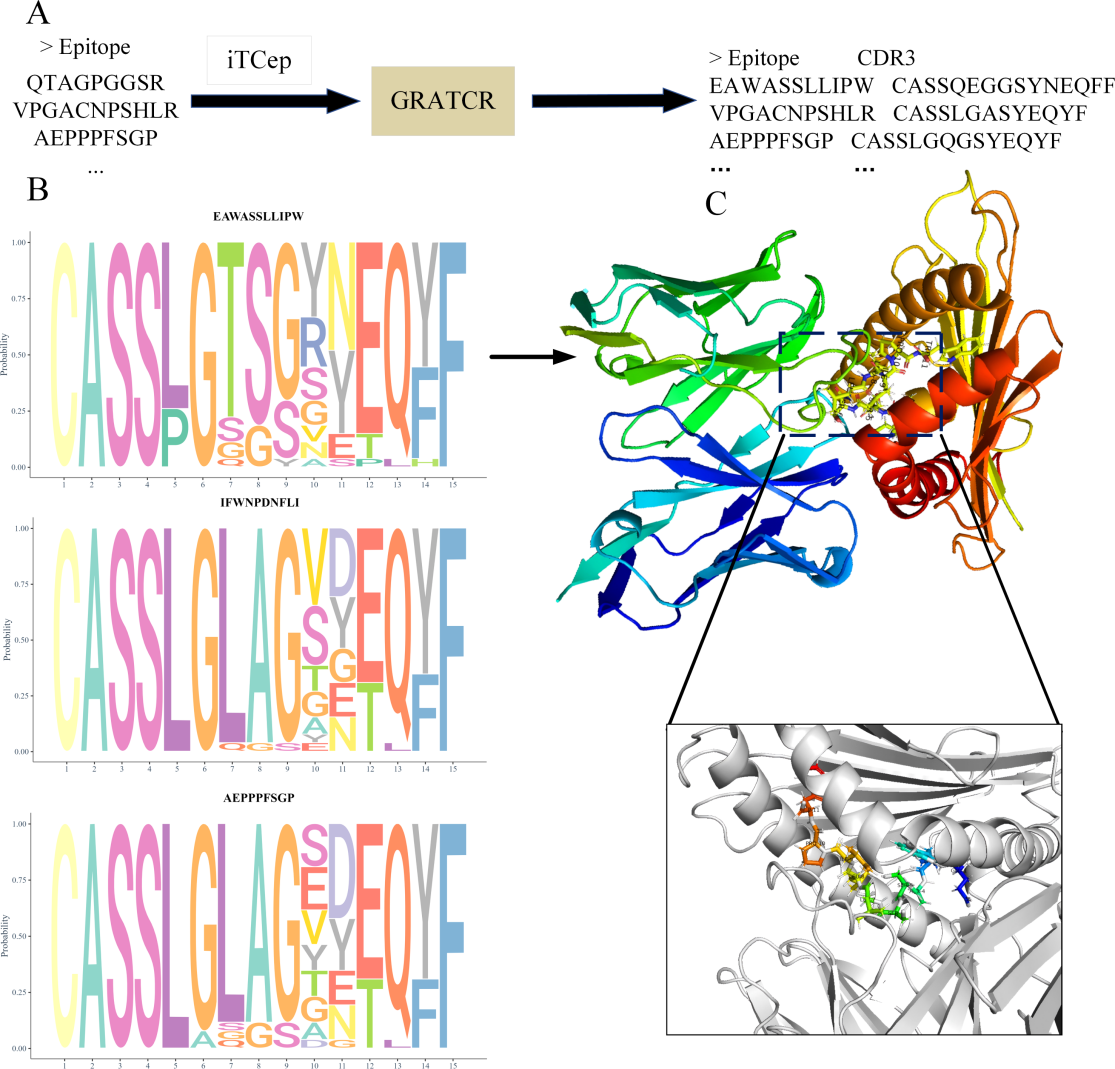
3D docking simulation of pMHC molecules with generated TCRs. **(A)** Use iTCep and GRATCR to computationally generate TCR sequences targeting specific peptides. **(B)** Three epitopes were selected for conservative analysis of the generated TCR sequences. **(C)** TCRmodel2 predicts and optimizes the three-dimensional structure of the TCR-pMHC complex, with visualization of pMHC-TCR using PyMOL.

Understanding the structural basis of TCR-pMHC complexes is critical for elucidating TCR specificity, rationally designing TCR affinity, and advancing applications in vaccine design, autoimmunity, and cancer therapeutics. In this case, we constructed three-dimensional pMHC-TCR complex models. Two *de novo* prediction methods, iTCep and GRATCR, were employed to generate core CDR3 β sequences capable of recognizing the selected neoantigen epitopes. GRATCR integrates an Epitope-BERT epitope encoder with a TCR-GPT receptor generator, enabling end-to-end generation of high-quality TCR CDR3 β sequences specific to a given antigen epitope. In parallel, iTCep is a deep learning framework independently developed by our team, which leverages feature fusion strategies to identify T cell epitopes responsive to neoantigens. Motif analysis of the generated CDR3 β sequences (**Figures 6A, B**; **Supplementary Figure S4**) revealed a certain degree of amino acid conservation at key contact sites, suggesting potential functional relevance in antigen recognition. Full-length TCR ɑ/β chain sequences were assembled accordingly. We then used TCRmodel2 to predict the three-dimensional structure of the TCR-pMHC complexes and visualized the resulting conformations using PyMOL. TCRmodel2 is a structure prediction tool based on the AlphaFold framework that accurately models both global topology and interfacial residue interactions. As an example, for the neoantigenic peptide EAWASSLLIPW, the predicted model exhibited a high local structure confidence score (pLDDT) of 92.66%, along with favorable global (pTM) and interfacial (ipTM) scores. The composite model confidence score reached 0.89, indicating a high likelihood that the predicted conformation closely approximates the true biological binding mode (**Figure 6C**, **Supplementary Table S3**). This structure provides a crucial spatial framework for elucidating the mechanism of TCR recognition of neoantigens. The resulting TCR-pMHC complex models offer theoretical support and potential targets for the development of TCR-engineered cellular therapies.

## Discussion

OmniNeo is a multi-omics, AI-driven based pipeline for neoantigen discovery and prioritization, designed to enhance the accuracy of neoantigen prediction and improve clinical applicability. The pipeline was applied to 4 patients with hepatocellular carcinoma, and 31 high-quality immune targets were effectively screened out by combining key immune features and deep learning model, and its effectiveness was proved by *in vitro* computational simulation. These targets were validated through *in vitro* simulations and hold potential for use in mRNA vaccines, peptide-based vaccines, and neoantigen-specific T cell immunotherapy.

Clinical studies have demonstrated that personalized neoantigen-based therapies have shown significant efficacy across various tumor types. Our group has previously developed a series of neoantigen prediction pipelines (11, 15, 18), each focusing on neoantigen products derived from specific mutation types, thereby laying the groundwork for systematic analysis of neoantigen origins. To further enhance the potential clinical application of neoantigen burden, we developed NAIRscore (63), which comprehensively integrates neoantigen load, HLA-I score and cytotoxicity score and can be effectively used for stratified assessment of patients’ immune response capacity and prognostic potential, and it was proved to be very useful and welcomed in multiple myoma patients (64). However, many immunogenic mutations tend to be low in frequency, or rare, or non-canonical, resulting in limited therapeutic benefit across patient populations. To comprehensively identify neoantigens arising from diverse mutation types, researchers often need to run multiple independent tools in parallel (e.g., pipelines targeting coding and non-coding regions). However, these tools lack standardization and integration, with inconsistencies in data formats, input requirements, and evaluation metrics. This fragmentation of workflows results in increased operational complexity, information loss during pipeline transitions, and reduced reproducibility of predictions. Therefore, there is an urgent need to develop a unified, high-throughput, and modular framework for neoantigen identification.

Here, we introduced OmniNeo, a workflow developed upon the foundation of our prior research and integrated resource platforms, designed to overcome the limitations of existing tools. In addition to identifying neoantigen candidates at the genomic and transcriptomic levels, OmniNeo incorporates proteomics-based filtering mechanisms, enhancing the reliability of candidate neoantigens. The workflow further integrates an AI-driven immunogenicity optimization module, producing high-quality and comprehensive neoantigen candidates suitable for the design of mRNA vaccines, peptide vaccines, and T cell therapies, offering technical support and practical examples for potential preclinical or clinical applications. Furthermore, OmniNeo is implemented as a fully integrated and streamlined neoantigen analysis workflow based on the nextflow framework. It offers portability, usability, and scalability, supporting batch processing of raw sequencing data and checkpoint resume functionality. This makes it particularly well-suited for high-throughput analyses involving multi-center and multi-patient cohorts. OmniNeo demonstrates substantial application potential and versatility, supporting multifaceted analyses across diverse clinical contexts.

Although the OmniNeo pipeline has broad application potential in cancer immunotherapy, its limitations exist for future further improvement. First, while OmniNeo integrates multi-omics data, its predictive accuracy remains dependent on the quality and depth of input data provided by users. In this study, due to the limited availability of matched sequencing data for hepatocellular carcinoma samples, conventional LC-MS/MS data were used to validate the protein-level expression of candidate antigenic peptides. Notably, the mass spectrometry module in OmniNeo is highly flexible and can be directly replaced to incorporate more sensitive immunopeptidomics data, if available, to capture evidence of HLA-mediated antigen presentation and further enhance the accuracy and biological relevance of neoantigen prediction. Second, although the workflow employs deep learning models to estimate the immunogenicity of neoantigen peptides, it lacks evaluation from the T cell perspective, particularly the activation status of immune cells (65). Additionally, OmniNeo has currently only verified its effectiveness in limited samples and specific tumor types, systematic testing and optimization in larger-scale clinical datasets are urgently needed to support its further translation and application in cancer immunotherapy.

Single-cell RNA sequencing (scRNA-seq) has provided new insights into the immune mechanisms underlying neoantigen recognition. Liu et al. integrated scRNA-seq with artificial intelligence models to elucidate T cell immune responses from a multimodal perspective and achieved precise identification of tumor-reactive neoantigens (66). TCellSI further combined scRNA-seq and bulk RNA-seq data to systematically characterize T cell functional states and visualize immune response dynamics (67). These advancements offer valuable references for identifying highly immunogenic neoantigens and optimizing vaccine design. In future work, we plan to incorporate scRNA-seq and other high-resolution datasets to refine the neoantigen prediction framework, while elucidating immune response mechanisms from both T cell activation and antigen generation perspectives. Finally, we will apply the optimized OmniNeo pipeline to a larger cancer patient cohort to improve its robustness and clinical applicability.

The development of the OmniNeo pipeline provides strong support for the design and implementation of neoantigen-based vaccines and TCR-T cell precision immunotherapies, facilitating the advancement of cancer vaccine research and clinical translation. In fact, high-quality candidate peptides have been synthesized *in vitro*, and will soon carry out subsequent target verification to highlight its clinical application potential.

## Code availability

OmniNeo is publicly available at https://github.com/linfengxu/OmniNeo; https://zenodo.org/records/15340824

## Data Availability

The original contributions presented in the study are included in the article/**Supplementary Material**. Further inquiries can be directed to the corresponding authors.
