# Supplementary material for: OmniNeo: a multi-omics pipeline incorporating proteomics and AI selection for neoantigen optimization in tumor immunotherapy
Source: Front Immunol. 2025 Dec 17;16:1727642. doi: 10.3389/fimmu.2025.1727642 (PMC12753916; doi:10.3389/fimmu.2025.1727642)
Supplement: Supplementary file 1 [file DataSheet1.docx]

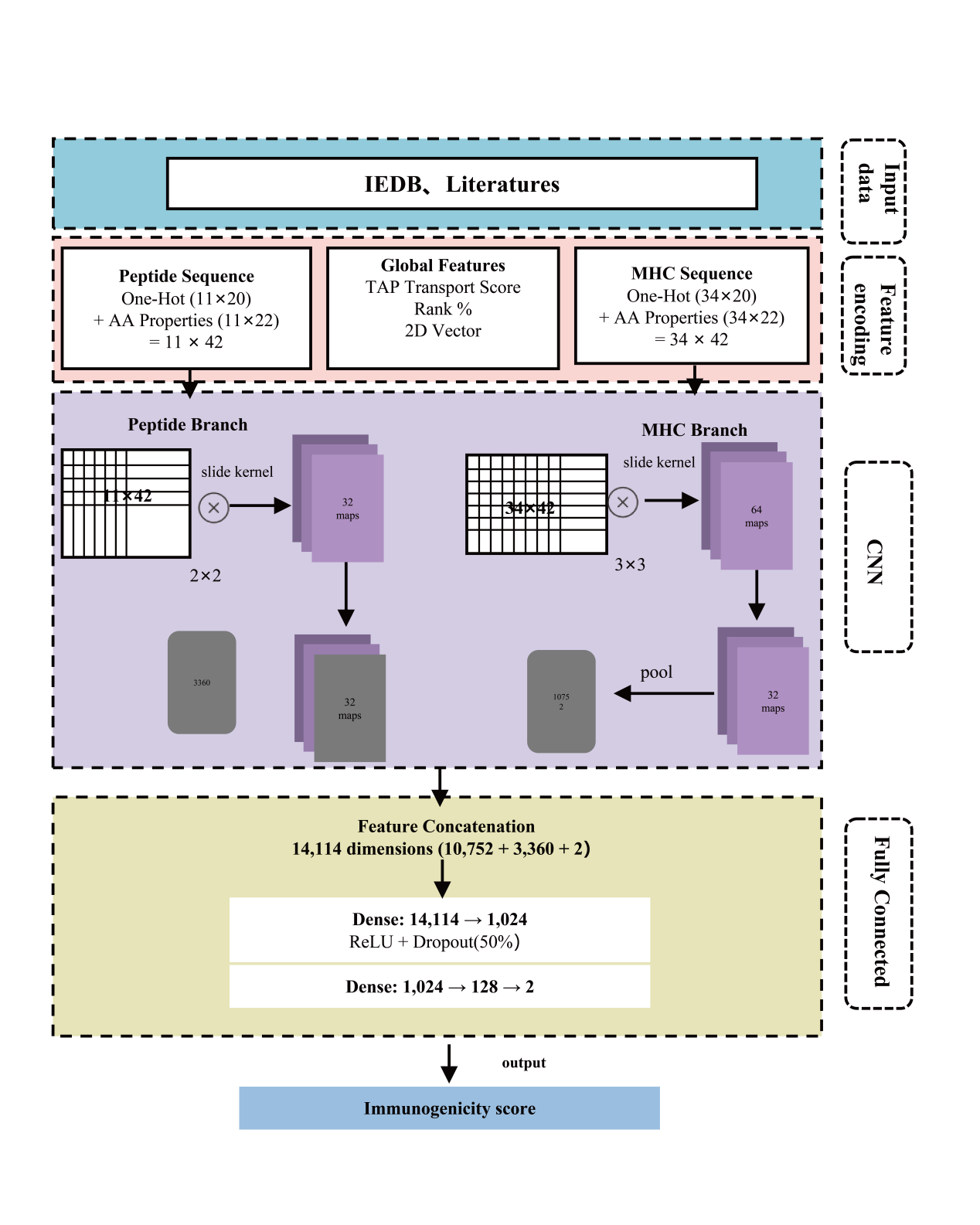


Figure S1. OmniNeo-CNN: a neural network-based model for predicting neoantigen immunogenicity.


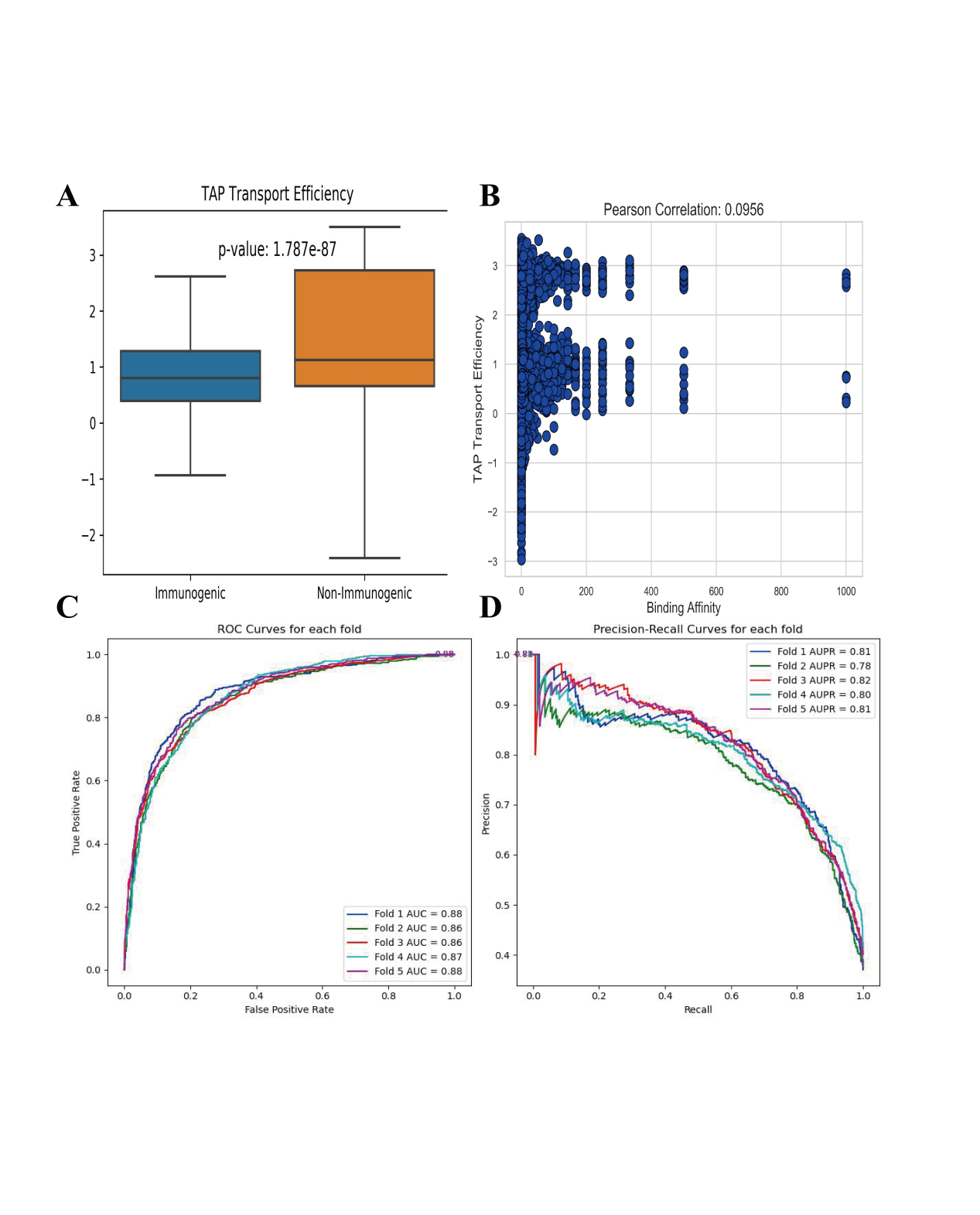


Figure S2. Feature selection in neoantigen epitopes and ROC curves of antigen epitopes classification. (A) Comparison of TAP transport efficiency distribution between immunogenic and non-immunogenic peptides. (B) Correlation between TAP transport efficiency and HLA binding affinity. (C) ROC curves of the classifiers for immunogenic prediction in the test dataset. (D) The Precision-Recall curves, with AUPR values ranging from 0.78 to 0.82, indicate that the model exhibits strong performance across all folds.


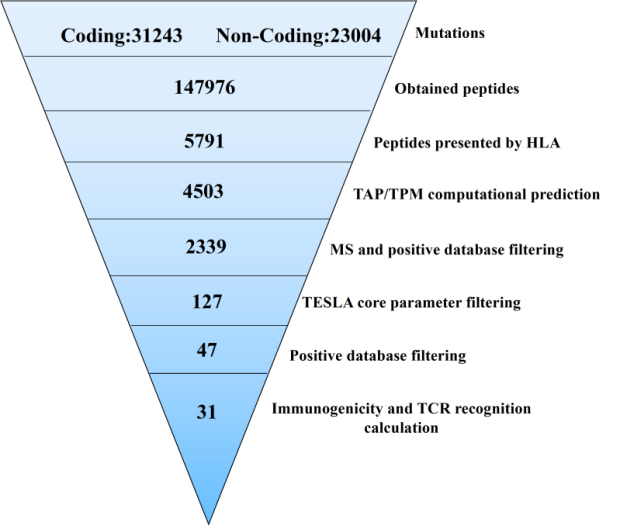


Figure S3. The process of neoantigen discovery based on the OmniNeo workflow. After successive filtering steps, 31 high-confidence peptides were ultimately retained to support subsequent vaccine development.


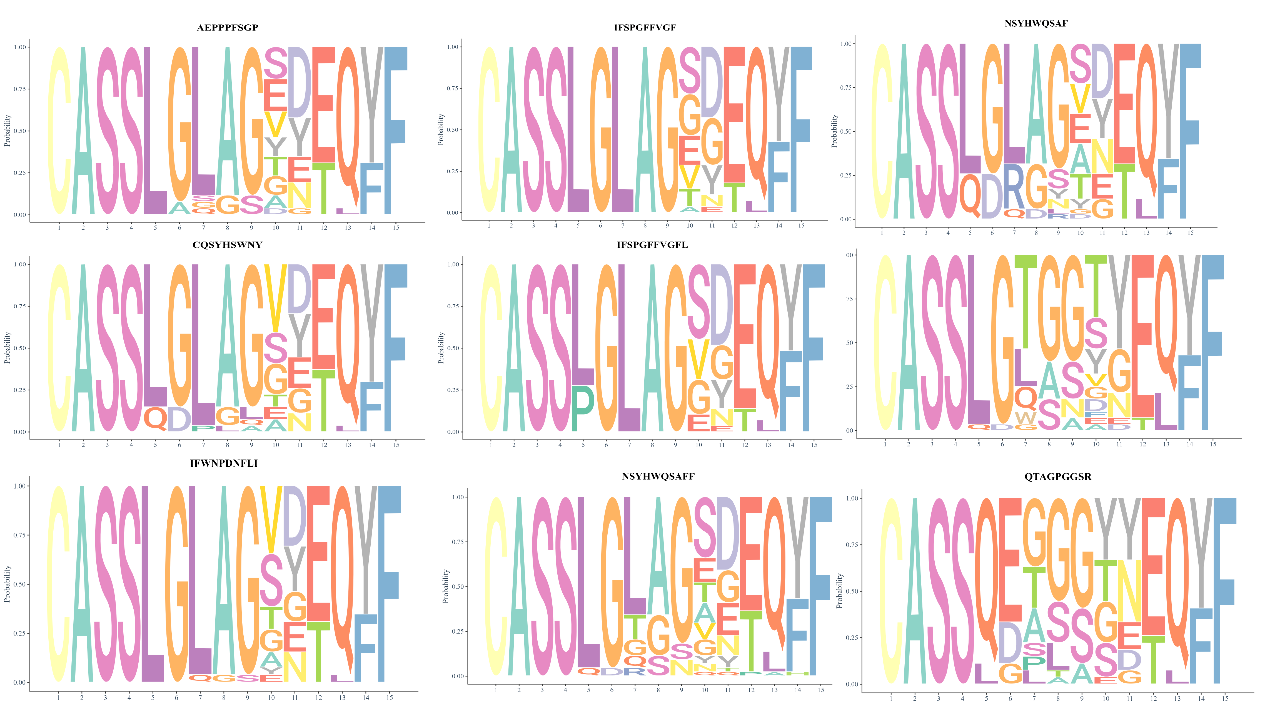


Figure S4. Motif analysis of the CDR3 β sequences potentially reactive to high-confidence peptides revealed distinct conservation patterns at key amino acid contact site

Table S1. The high-confidence neoantigen list for four HCC patients obtained through the OmniNeo workflow.

| **Patient ID** | **Peptide number** | **Gene** | **Peptide** | **HLA allele** | **%Rank_EL** | **Bind Level** | **Tap_prediction_score** | **Tpm** | **Predict score** |
| --- | --- | --- | --- | --- | --- | --- | --- | --- | --- |
| L052 | 77 | SNRPD3 | QTAGPGGSR | HLA-A*33:03 | 0.164 | SB | 1.625 | 36.4233 | 0.99980897 |
| L052 | 110 | PRAP1 | VPGACNPSHLR | HLA-A*33:03 | 1.589 | WB | 1.211 | 199.057 | 0.9993376 |
| L052 | 4 | CHST2 | AEPPPFSGP | HLA-B*40:01 | 1.47 | WB | 0.146 | 59.5435 | 0.9991462 |
| L052 | 17 | SDC4 | FFPSSPPNK | HLA-A*33:03 | 1.707 | WB | 0.654 | 474.916 | 0.9987803 |
| L052 | 16 | ITIH1 | EAWASSLLIPW | HLA-B*58:01 | 0.464 | SB | 0.849 | 79.0796 | 0.9987172 |
| L052 | 13 | NUB1 | CSSHVTEAL | HLA-C*03:04 | 1.161 | WB | 0.845 | 47.4322 | 0.9966794 |
| L041 | 81 | ZBTB20 | SACQSYHSWNY | HLA-B*15:01 | 1.58 | WB | 2.987 | 43.0351 | 0.99634176 |
| L056 | 34 | CXCL10 | HSDLLPYL | HLA-A*02:07 | 1.946 | WB | 0.737 | 104.043 | 0.99607486 |
| L052 | 95 | SNRPD3 | TAGPGGSRL | HLA-C*03:02 | 0.576 | WB | 0.771 | 36.4233 | 0.99591184 |
| L048 | 87 | LAMP2 | SIFWNPDNFLI | HLA-A*23:01 | 1.993 | WB | 0.837 | 33.138 | 0.9953564 |
| L041 | 3 | ZBTB20 | ACQSYHSWNY | HLA-B*15:01 | 0.77 | WB | 3.111 | 43.0351 | 0.99258196 |
| L056 | 90 | MRPL30 | SSLGDRMRL | HLA-C*01:02 | 1.857 | WB | 1.114 | 36.9893 | 0.9902964 |
| L052 | 37 | NUB1 | IFSPGFFVGFL | HLA-A*24:02 | 0.967 | WB | 1.036 | 47.4322 | 0.98738444 |
| L052 | 36 | NUB1 | IFSPGFFVGF | HLA-A*24:02 | 0.119 | SB | 2.582 | 47.4322 | 0.9824112 |
| L048 | 38 | LAMP2 | IFWNPDNF | HLA-A*23:01 | 0.875 | WB | 2.945 | 33.138 | 0.98022974 |
| L048 | 39 | LAMP2 | IFWNPDNFL | HLA-A*02:07 | 1.614 | WB | 1.365 | 33.138 | 0.97736174 |
| L048 | 86 | LAMP2 | SIFWNPDNF | HLA-A*23:01 | 1.339 | WB | 2.837 | 33.138 | 0.9744493 |
| L048 | 41 | LAMP2 | IFWNPDNFLIF | HLA-A*23:01 | 0.175 | SB | 2.906 | 33.138 | 0.9701531 |
| L056 | 83 | LAMP2 | SAPLHSSL | HLA-C*01:02 | 0.009 | SB | 1.093 | 67.0973 | 0.9672133 |
| L048 | 40 | LAMP2 | IFWNPDNFLI | HLA-A*23:01 | 0.875 | WB | 0.951 | 33.138 | 0.9430501 |
| L052 | 25 | NUB1 | FSPGFFVGF | HLA-A*24:02 | 0.849 | WB | 2.474 | 47.4322 | 0.9214925 |
| L052 | 14 | NUB1 | DIFSPGFFVGF | HLA-A*24:02 | 1.792 | WB | 2.354 | 47.4322 | 0.9159003 |
| L052 | 59 | ITIH1 | LLIPWGSKM | HLA-C*03:02 | 0.433 | SB | 0.445 | 79.0796 | 0.910154 |
| L048 | 115 | LAMP2 | WNPDNFLIF | HLA-A*23:01 | 1.831 | WB | 2.235 | 33.138 | 0.8980407 |
| L041 | 12 | ZBTB20 | CQSYHSWNY | HLA-B*15:01 | 0.614 | WB | 3.109 | 43.0351 | 0.8675678 |
| L041 | 74 | ZBTB20 | QLWYDWQAL | HLA-A*02:01 | 0.941 | WB | 1.054 | 43.0351 | 0.8360288 |
| L041 | 75 | ZBTB20 | QLWYDWQALV | HLA-A*02:01 | 0.392 | SB | 0.414 | 43.0351 | 0.79646724 |
| L056 | 67 | ATRN | NSYHWQSAF | HLA-B*46:01 | 0.123 | SB | 2.847 | 49.1498 | 0.78334427 |
| L056 | 68 | ATRN | NSYHWQSAFF | HLA-B*46:01 | 1.957 | WB | 2.694 | 49.1498 | 0.78334427 |
| L041 | 20 | ZBTB20 | FQLWYDWQAL | HLA-A*02:01 | 1.802 | WB | 0.901 | 43.0351 | 0.5746423 |
| L041 | 118 | YWHAZ | YAKILNGTHY | HLA-B*15:01 | 0.57 | WB | 3.003 | 44.3763 | 0.57334244 |

Table S2. Experimentally validated neoantigenic sites in four cancer samples.

| Tumor type | Patient ID | Validated position |
| --- | --- | --- |
| Melanoma | 3998 | MAGEA6-E168K  PDS5A-Y1000F; H1007Y  MED13-P1691S |
|  | 3784 | FLNA-R2049C  KIB16B-L1009P  SON-R1927C* |
|  | 3903 |  |
| Gastrointestinal tumor | 3942 | GPD2-E426K  NUP98-A359D  KARS-D328H |

Table S3. The model parameters refined. Model.

| PDB | pLDDT | pTM | ipTM | TCR-pMHC ipTM | l-pLDDT | Model confidence |
| --- | --- | --- | --- | --- | --- | --- |
| **ranked_0** | **92.66** | **0.9** | **0.89** | **0.87** | **77.68** | **0.89** |
| ranked_1 | 91.86 | 0.85 | 0.81 | 0.75 | 76.05 | 0.82 |
| ranked_2 | 91.07 | 0.85 | 0.81 | 0.77 | 75.98 | 0.82 |
| ranked_3 | 91.54 | 0.84 | 0.81 | 0.75 | 74.38 | 0.81 |
| ranked_4 | 86.49 | 0.52 | 0.36 | 0.15 | 67.6 | 0.39 |

confidence >= 0.85 - model likely good. Model confidence <= 0.49 - model likely not good.
